# The Monthly Medical Review
†Feb. 13, 1891.


**Published:** 1891-06-06

**Authors:** 


### THE PRACTITIONER'S BOOKSHELF.

### "THE MONTHLY MEDICAL REVIEW."†

With the inevitable tendency to specialism in practice, as a result of the growth of medical science, it is only natural that we should find a corresponding growth and subdivision in medical literature. We have before us an early number of *The Monthly Medical Review, a general practitioner's journal.* As the title implies, it is intended to meet the special wants of the general practitioner. The first ten pages are occupied with "monthly notes" and notices. The next twelve pages are devoted to "current medical politics," and this should prove an interesting and useful section. We are glad to welcome a contemporary which recognises the necessity for devoting time and space to medical politics, although the opinions expressed do not always accord with our own. The remaining pages are occupied by abstracts and selections from medical literature, notes on new preparations, &c.

† Feb. 13, 1891.

